# The Role of the Nrf2 Pathway in Airway Tissue Damage Due to Viral Respiratory Infections

**DOI:** 10.3390/ijms25137042

**Published:** 2024-06-27

**Authors:** Arnaud John Kombe Kombe, Leila Fotoohabadi, Ravikanth Nanduri, Yulia Gerasimova, Maria Daskou, Chandrima Gain, Eashan Sharma, Michael Wong, Theodoros Kelesidis

**Affiliations:** 1 Division of Infectious Diseases and Geographic Medicine, Department of Internal Medicine, University of Texas Southwestern Medical Center, Dallas, TX 75390, USA; arnaud.kombekombe@utsouthwestern.edu (A.J.K.K.);; 2 Division of Infectious Diseases, Department of Medicine, David Geffen School of Medicine, University of California Los Angeles, Los Angeles, CA 90095, USA

**Keywords:** respiratory virus, Nrf2, virus-induced injury, lung injury, inflammation, pyroptosis, apoptosis, ferroptosis, fibrosis, autoimmunity, oxidative stress

## Abstract

Respiratory viruses constitute a significant cause of illness and death worldwide. Respiratory virus-associated injuries include oxidative stress, ferroptosis, inflammation, pyroptosis, apoptosis, fibrosis, autoimmunity, and vascular injury. Several studies have demonstrated the involvement of the nuclear factor erythroid 2-related factor 2 (Nrf2) in the pathophysiology of viral infection and associated complications. It has thus emerged as a pivotal player in cellular defense mechanisms against such damage. Here, we discuss the impact of Nrf2 activation on airway injuries induced by respiratory viruses, including viruses, coronaviruses, rhinoviruses, and respiratory syncytial viruses. The inhibition or deregulation of Nrf2 pathway activation induces airway tissue damage in the presence of viral respiratory infections. In contrast, Nrf2 pathway activation demonstrates protection against tissue and organ injuries. Clinical trials involving Nrf2 agonists are needed to define the effect of Nrf2 therapeutics on airway tissues and organs damaged by viral respiratory infections.

## 1. Introduction

Respiratory viral infections, such as influenza and coronavirus disease 2019 (COVID-19), cause significant mortality and morbidity worldwide. Respiratory viruses (RVs) commonly affect respiratory tract airways. They are classified as upper respiratory tract infection (URTI)-associated injuries, which are commonly mild and transient, and lower respiratory tract infection (LRTI)-related injuries, which are commonly severe and associated with chronic diseases and mortality. Up to 70% of pneumonia cases are attributed to viral infections [1,2,3]. Acute respiratory distress syndrome (ARDS) is characterized by diffuse lung inflammation and edema, hypoxemia, and acute respiratory failure and is commonly caused by viral pneumonia. Of note, more than 4 million deaths are attributed to virus-induced LRTI-associated injuries [4].

The RVs that cause ARDS-induced pneumonia include severe acute respiratory syndrome (SARS)-CoV (2002) [5], H5N1 and H1N1 influenza viruses (2009) [5,6], MERS-CoV (2012), and SARS-CoV-2 (2019) [7,8], which is the leading cause of the COVID-19 pandemic. Furthermore, other lower respiratory tract infections, including bronchitis and bronchiolitis, are caused by RVs, especially respiratory syncytial viruses (RSVs), influenza viruses (IVs), parainfluenza (PIV), and adenoviruses, which are more diagnosed in children and infants with bronchitis and bronchiolitis [9,10]. Human rhinoviruses (HRVs), PIVs, RVS, CoVs, adenoviruses, and IVs are the predominant etiological agents of upper respiratory tract infections (URTIs). Several host factors drive viral-induced tissue damage during respiratory viral infections. However, it remains unclear how RVs interact with host tissue responses.

During infection, RVs induce the generation of reactive oxygen and nitrogen species (ROS and RNS), which disrupt redox homeostasis and subsequently lead to oxidative stress [11,12]. Viruses that induce prooxidant cellular responses include (hMPV) [13], HRVs [14,15,16,17], enteroviruses (EVs, including EV-71 and Coxsackievirus B3 [18,19,20] CoVs [21,22,23,24,25,26,27,28,29], RSVs [13,30,31,32,33,34,35,36,37,38,39,40,41,42], IVs [43,44,45,46,47,48,49,50], and PIVs [51,52]. Increased viral-induced oxidative stress then compromises the infected host cells through different molecular and cellular structure damages and increased viral replication, altering the function of organs and the immune response field [12,53,54]. Of note, increased redox stress is the main leading cause of respiratory virus-induced injuries and diseases. To clear viral infection(s) and reestablish cellular redox balance, the host deploys a solid and specific antiviral response, especially the antioxidant host response, which is mainly modulated by the activated nuclear factor erythroid 2-related factor 2 (Nrf2) pathway.

**Figure 1:**
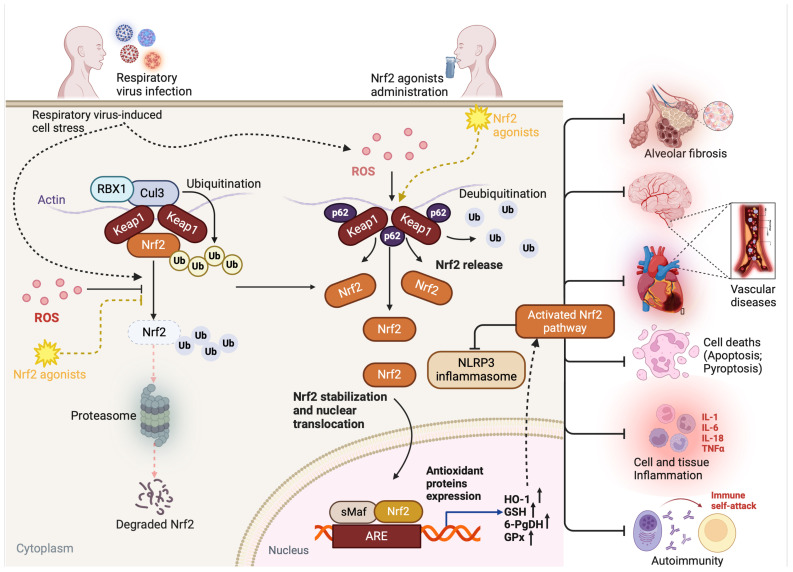
The role of the Nrf2 pathway in airway injuries due to viral respiratory infections. Viral respiratory infections are one of the main leading causes of airway damage. Respiratory virus-associated injuries include oxidative stress, ferroptosis, inflammation, pyroptosis, apoptosis, fibrosis, autoimmunity, and vascular injury. The inhibition or deregulation of Nrf2 pathway activation induces airway tissue damage in viral respiratory infections. The inhibition or downregulation of the Nrf2 pathway can be rescued by Nrf2 agonists that boost the activation of Nrf2. (Created with BioRender.com, 2024, accessed on 12 June 2024).

Nrf2 is a protein capable of binding to the nuclear factor, the erythroid-derived 2/activator protein 1 (NF-E2/AP1) repeat of the beta-globin gene [55]. After discovering its beneficial role in regulating the expression of many antioxidant and detoxification enzymes, including heme oxygenase-1 (HO-1) and glutathione S-transferases (GSTs), Nrf2 became the target and center of extensive research. Since then, it has been demonstrated that Nrf2 regulates balance and maintains the homeostasis of the cellular redox system by controlling the production level of ROS and the activation of antioxidant immune molecules (HO-1 and GSTs). Specifically, antioxidant treatments [37] and host-secreted cytosolic HO-1 and GSTs have been associated with a relevant decrease in respiratory viral-associated oxidative stress-induced cell damage and anti-inflammatory and anti-apoptotic properties [47,56,57,58], which soothe respiratory virus-associated infections and damage and attenuates complications (Figure 1).

As we recently reviewed, the Nrf2 pathway also plays an essential role in the replication of respiratory viruses and helps to restore disrupted redox homeostasis. However, the way in which viral replication interacts with the Nrf2 pathway is complex since RVs can leverage the Nrf2 pathway for their replication. Independent of the effects on viral replication, Nrf2 may play protective roles in cell and tissue damages induced by RVs [45]. Non-exhaustively, the cell tissues and organs affected in viral pathogenesis include the pharynx, larynx, trachea, and, most importantly, lung cells and tissues, for which severe damage is associated with respiratory distress and life-threatening pathologies. However, the role of Nrf2 pathways in cell and tissue damage remains unclear since both the downregulation/inhibition or upregulation/hyper-activation of this pathway can have complex effects on several host pathways that include but are not limited to cell inflammation, apoptosis, ferroptosis, fibrosis, vascular dysfunction, and autoimmunity [59,60,61,62]. Herein, we review the scientific evidence regarding the critical role of the Nrf2 pathway in the pathogenesis of end-organ airway tissue damage associated with RVs with a focus on common viruses such as HRVs, RSVs, IVs, and CoVs (SARS-CoV-2). Understanding host pathways mediating virus-induced tissue damage may lead to the development of novel therapies for airway damage induced by viral respiratory diseases.

## 2. The Nrf2 Pathway

Activating NRF2 enables cells to adapt to stress reactions and maintain redox balance. Mechanistically, Nrf2 is first synthesized in the cytoplasm and then combines with Keap1 through the Neh2 domain of Nrf2 that can recognize different sites in Keap1 and form the disulfide-bounded Nrf2-Keap1 complex [63,64].

Without triggers or infection, the Nrf2 signaling cascade remains inactive. In the Nrf2-Keap1 complex, cysteine residue contributes to the transfer of dipeptide derivatives, enabling the ubiquitination and degradation of Nrf2 through the proteasome signaling pathway, which actively processes to maintain Nrf2 at a low cytoplasmic level (Figure 1).

Several signaling cascades have been proven to activate the Nrf2 pathway under stress, including the p239/MDM2 pathway, which requires a complex collaboration to allow for the normal activation of Nrf2. However, upon infection, cells produce enough harmful factors, which trigger a series of reactions, causing excessive cytosolic ROS and RNS production and specifically inducing the disruption of the internal balance of oxidation and antioxidants (redox homeostasis). Notably, during infection, the synthesis levels of superoxide (O_2_^•−^)-, hydroxyl (HO^•^)-, peroxyl (RO^•−2^)-, and hydroperoxyl (HO^•2^)-derived ROS, nitric oxide (NO)-derived RNS, the transcription of the inducible NO synthase (iNOS) gene, and the phosphorylation of STAT-1 significantly increase as they are beneficial for host defense mechanisms and pathogen clearance [65,66,67]. However, because of the cytotoxicity of these molecules for tissues and organs, in abnormally high concentrations, they may induce inflammation, allergic and autoimmune diseases, and multiple types of damage in the airways when they are not well regulated or upon persistence of the infection [65,66]. Thus, under these infection-induced stress conditions, Keap1 undergoes oxidative modification, leading to a conformational change in the shape of the Keap1-Nrf2 complex, releasing Nrf2 from Keap1, which accumulates in the cytoplasm. Cytosolic accumulated Nrf2 translocates into the nucleus and then binds to the antioxidant response element (ARE) to initiate the transcription of various cytoprotective genes (Figure 1).

When Nrf2 reaches the nucleus, it activates the production of ROS-quenching genes to rebalance the production and scavenging of ROS. These stressors induce the production of a group of cytoprotective antioxidant enzymes, including HO-1, NAD(P)H quinone oxidoreductase 1 (NQO1), superoxide dismutases (SOD), catalase, glutathione peroxidase (GPx), glutathione reductase, GST, thioredoxins, γ-glutamyl-cysteine ligase (γ-GCL), and many isoforms of aldo-keto reductases, by using Nrf2 to recognize the ARE promoter (Figure 1) [68,69]. The number of NRF2-regulated genes is extensive and includes the most protective antioxidants, Phase II detoxifying enzymes, and many drug influx/efflux transporters that can serve to eliminate both endogenous and exogenous toxic products [68,70].

This activation of the Nrf2 signaling cascade is crucial in establishing cell redox homeostasis, defense against RVs, and the pathogenesis of related respiratory diseases. As we recently reviewed, the activated Nrf2 pathway also plays an essential role in the replication of respiratory viruses. Herein, we will review the scientific evidence regarding the role of the Nrf2 pathway in respiratory virus-induced oxidative stress and ferroptosis, inflammation and pyroptosis, apoptosis, fibrosis, autoimmunity, and vascular injury, which collectively drive tissue damage and end-organ disease.

## 3. The Role of the Nrf2 Pathway in Respiratory Virus-Induced Oxidative Stress and Ferroptosis

As outlined above, oxidative stress has a significant role in the pathogenesis of respiratory viral infections. One aspect important for redox-related pathogenesis is ferroptosis, a programmed and regulated cell death mechanism used by host cells to clear infections by pathogens, including RVs. Discovered a decade ago and unlike other programmed cell deaths, ferroptosis is an exclusively iron-dependent process characterized and induced by the excessive production and accumulation of iron-mediated ROS and lipid peroxides [71]. Ferroptosis cell death differs from autophagy and other programmed cell deaths, such as apoptosis and pyroptosis, at different levels, including at the morphological, biochemical, and genetic levels (reviewed in [71]). Infections with RVs have been associated with several pathophysiological signatures of ferroptosis that drive respiratory tissue and organ damage, including the disruption of the cell redox homeostasis and depletion of glutathione peroxidase 4 (GPX4) and antioxidant glutathione (GSH) (Table 1) [72,73,74,75,76,77,78,79,80,81,82,83,84,85,86,87,88,89,90].

**Figure 2:**
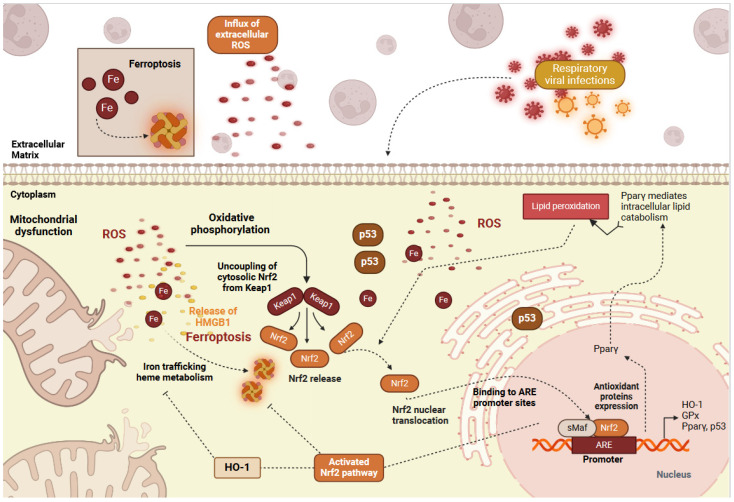
The cross-talk between the Nrf2 pathway, oxidative stress, and ferroptosis in viral respiratory infections. Networks behind the formation of ferroptosis and subsequent Nrf2 activation during viral respiratory infections. Ferroptosis is primarily characterized by iron (Fe) accumulation and the formation of iron complexes. Iron and ROS accumulation sensitize the cellular membrane to exhibit PPARγ-mediated lipid peroxidation, thereby activating Nrf2. The activation of the Nrf2 pathway inhibits ferroptosis through the transcription of heme-oxygenase enzyme HO-1 and glutathione synthesis enzymes GPx and Gclc, as well as the regulation of p53 and PPARγ. Increased cytosolic HMGB1, ROS, and iron concentrations also lead to mitochondrial dysfunction. ROS, reactive oxygen species; GSH, reduced glutathione; GSSG, oxidized glutathione; GPx, glutathione peroxidase; Nrf2, nuclear factor erythroid 2-like factor 2; Keap1, Kelch-like ECH-associated protein 1; Pparγ, peroxisome proliferator-activated receptor gamma; HMGB1, high mobility group box 1; HO-1, heme-oxygenase-1; Gclc, γ-glutamyl cysteine ligase; ARE, antioxidant response element. (Created with BioRender.com, 2024, accessed on 12 June 2024).

Nrf2 transcriptionally regulates most of the genes involved in ferroptosis, such as GPX4 [93,94,95] (Figure 2). Recently, different RVs have been shown to inhibit ferroptosis by inducing Nrf2 degradation. H1N1 virus infection caused the differential expression of many ferroptosis-related genes and metabolites in human nasal mucosal epithelial cells by suppressing the expression of the glutamate-cysteine ligase catalytic (GCLC) subunit via the Nrf2 -KEAP1 pathway [96]. Tripartite motif-containing protein 21 (TRIM21) has been shown to physically interact with SQSTM1 and Nrf2 -KEAP1 and mediates Nrf2 degradation to induce oxidative stress and ferroptosis during pathogenic avian IV H5N1 infection [97]. Pyrogallol, a natural polyphenol compound (1,2,3-trihydroxybenzene), promoted the expression and nuclear translocation of Nrf2 and alleviated influenza A disease in human alveolar epithelial cells and mice [98]. When SARS-COV2 spike protein was overexpressed in HEK293 cells, Nrf2 showed an upregulation of Nrf2 in the presence of palmitic acid (PA) overload [99]. SARS-CoV-2 ORF3a promotes the degradation of Nrf2 through recruiting Keap1, thereby attenuating cellular resistance to oxidative stress and facilitating cells to ferroptotic cell death [100,101]. These data suggest that the cross-talk between the Nrf2 pathway and ferroptosis is essential for the pathogenesis of viral respiratory infections [102].

## 4. The Role of the Nrf2 Pathway in Respiratory Virus-Induced Inflammation and Pyroptosis

The airway epithelium consists of the large physical inner body surface barrier that directly interacts with and senses antigens and infectious agents to initiate immune responses. The immune response is triggered to clear the infection, but the whole mechanism requires the epithelium to undergo injuries (at least minor) characterized by epithelial cell inflammation and death. Even though the lower airway cells and tissues are sites where inflammations occur in most respiratory viral infections, the upper airway remains the first point of contact with RVs, and thus, the prime site of tissue inflammation, such as sinusitis, laryngitis, pharyngitis, and, ultimately, lung inflammation. Mechanistically, upon active respiratory viral infection, viral particles infiltrate the upper airways and spread across the lower airway epithelial cells, triggering the host’s innate and adaptive immune responses. In immunocompetent people with healthy airway tracts, tissue inflammation and the pyroptosis of immune cells are beneficial and transient due to a robust and highly regulated type 1 inflammatory response, which leads to a clearance of the respiratory viral infection [103,104]. However, in people with unhealthy airways and compromised health conditions, the inflammatory response may be impaired and inefficient, exacerbating the viral replication and enhancement of the immune cell infiltration and lung disease [105]. Thus, short-term airway inflammation contributes to viral clearance and the healing process [59,60,61,106], while sustained inflammation is associated with severe injuries and the immunopathology of viral infection in airway tissues (Table 2).

**Figure 3:**
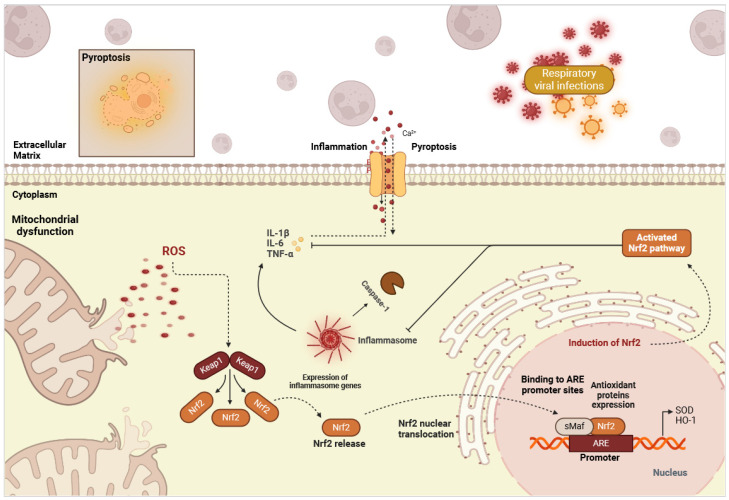
The cross-talk between the Nrf2 pathway, inflammation, and pyroptosis in viral respiratory infections. Antioxidant products increase Nrf2 activity, attenuating ROS production and preventing NF-kB oxidation. Some amino acid residues are oxidized in these compounds for inflammasome activation and pyroptosis induction. (Created with BioRender.com, 2024, accessed on 12 June 2024).

Known to regulate and maintain redox homeostasis, Nrf2 also significantly influences inflammation through various mechanisms (Figure 3). The activation of Nrf2 has been shown to trigger an anti-inflammatory response and concomitantly inhibit the up-regulation of pro-inflammatory cytokines like IL-6, IL-18, and IL-1β, likely through interference with NF-κB-mediated transcription [131]. This regulatory role extends to other inflammatory mediators, such as chemokines and cell adhesion molecules (CAMs), which are crucial for leukocyte recruitment and tissue inflammation and damage [132]. Moreover, Nrf2 influences the balance between proteases and antiproteases, thus modulating tissue remodeling and inflammatory responses. Additionally, Nrf2 signaling interacts with matrix metalloproteinases (MMPs), enzymes involved in extracellular matrix degradation, and (COX), which catalyze the formation of pro-inflammatory prostaglandins. The activation of Nrf2 has been linked to the suppression of MMPs and COX-2 expression, contributing to the resolution of inflammation [132]. Experimental evidence demonstrates that Nrf2-controlled HO-1 induction leads to anti-inflammatory effects, such as increased IL-10 release in M2 macrophages and the attenuation of inflammation in various RV-induced pathological conditions [132]. Furthermore, studies showed that Nrf2 activation suppresses the expression of pro-inflammatory proteins and cytokines like cyclooxygenases (COX-2), iNOS, TNF-α, IFN-γ, and IL-6. For instance, in Nrf2-deficient mice models, inflammatory responses were exacerbated [133].

Notably, in IV infections, Nrf2 activation reduces NF-κΒ-mediated inflammation and the associated lung permeability damage and mucus hypersecretion [48,49,50]. Interestingly, the protective effects of Nrf2 seem to extend to resolving inflammation in influenza-induced chronic airway inflammation [132,134,135]. RSV-induced bronchopulmonary epithelial injury and inflammation were higher in Nrf2 knock-out mice than in the control Nrf2^+/+^ mice. Nrf2 knock-out mice infected with RSV had reduced viral clearance, low IFN-gamma levels, decreased body weights, and dysregulated antioxidant activity [39,40,41,42,131]. Moreover, as inflammation is modulated by NLRP inflammasomes [136], the masterpiece of pyroptosis activation (Figure 3), pretreatment with sulforaphane or viroporin prevents NLRP3 inflammasome activation and significantly limits lung RSV/rhinovirus replication and NLRP3-induced inflammation in control Nrf2^+/+^ but not in Nrf2 knock-out mice, suggesting the pivotal role of Nrf2 in controlling virus infection [17,39,49] and specifically by hampering NLRP3 activation and associated inflammation [39,40,41,42,131,133]. This is also true in CoVs (SARS-CoV-2), rhinovirus, enterovirus 71, and metapneumovirus infections, where infected transgenic Nrf2^−/−^ or Keap1^−/−^ mice display antioxidant enzyme depletion, increased K^+^ efflux, oxidative stress, and virus severity-induced airway damage compared with Nrf2^+/+^ mice [13,18,26,28,29,137,138].

Taken together, Nrf2 exerts a multifaceted influence on inflammation and prevents aberrant and exacerbated inflammation by regulating various pathways and mediators involved in the inflammatory response. This intricate network highlights the therapeutic potential of targeting Nrf2 in inflammation associated with viral respiratory infections [33,139].

## 5. The Role of the Nrf2 Pathway in Apoptosis during Viral Respiratory Infections

Apoptosis is a physiologic process characterized by programmed cell death induced during reversible tissue expansion to control cell growth, replace unwanted old cells with new ones, and preserve tissues and organs [140]. Upon infection by viruses, for example, apoptosis is induced as a host-programmed death of virus-infected cells to fight viral infection. However, in most respiratory viral infections, apoptosis usually does not only serve as a protection mechanism for the host to eliminate the virus-infected cells but also, more commonly, promotes viral infection (Table 3).

Notably, during infections with RVs, apoptosis has been associated with and characterized by prolonged respiratory epithelial cell and airway tract tissue injuries because RVs synthesize viral particles that dysregulate apoptosis and promote the lytic cell cycle.

**Figure 4:**
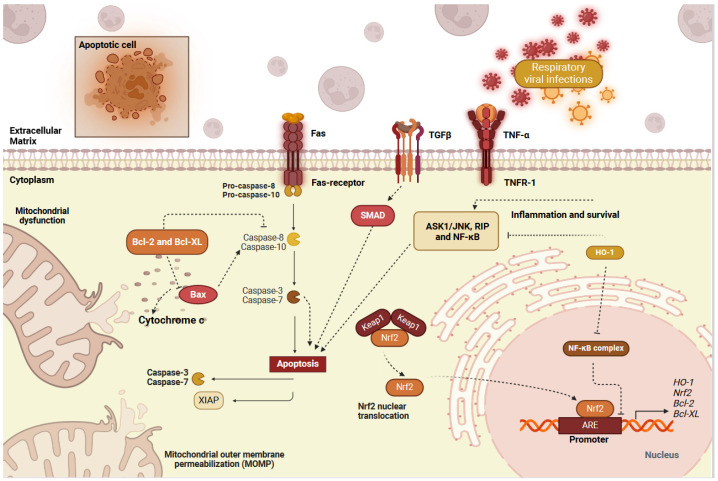
The cross-talk between the Nrf2 pathway and apoptosis in viral respiratory infections. The extrinsic induction of apoptosis occurs through cell surface receptor TGFβ, the downstream signaling of Smad, and the transduction of death domain receptors Fas and TNFα. Fas ligand binding to the receptor initiates the activity of cell execution molecules caspases 8/10, which subsequently activate pro-apoptotic caspases 3/7. Caspase 9 is an executioner molecule that intrinsically induces apoptosis and is activated by Bax-induced cytochrome c production. TNFα and downstream molecules transduce apoptotic signals, specifically ASK1/JNK, RIP, and NF-κB. The NF-κB complex hinders Nrf2 binding on promoter sites of target genes and repels it through Keap1 entry into the nucleus. As a response against apoptotic signaling, Nrf2 initiates the transcription of Bcl-2 and Bcl-XL to inhibit caspase signaling and HO-1 to limit NF-κB complex-mediated Nrf2 inactivation. TGFβ, tumor necrosis factor-beta; TNF-α, tumor necrosis factor-alpha; ASK1, apoptosis signal-regulating kinase 1; JNK, c-Jun N-terminal kinases; RIP, receptor-interacting protein; NF-κB, nuclear factor-κ of B cells; HO-1, heme-oxygenase-1; Bcl-2, B-cell lymphoma-2; Bcl-XL, B-cell lymphoma-extra-large; Bax, Bcl-2-associated X protein; Nrf2, atomic factor erythroid 2-like factor 2; Keap1, Kelch-like ECH-associated protein 1; ARE, antioxidant response element; Gclc, γ-glutamyl cysteine ligase. (Created with BioRender.com, 2024, accessed on 12 June 2024).

Many reports demonstrated that the activated Nrf2 pathway plays a crucial role and has been shown to modulate apoptosis in a bidirectional manner (Figure 4). Nrf2 anti-apoptotic effects are mainly attributed to its ability to induce anti-apoptotic protein BCL2 transcription [151]. Nrf2 over-expression has also been shown to activate ERK1/2 and its downstream target, ELK1, suppressing IL-1β-induced apoptosis [152]. Nrf2 protects against H1N1 influenza virus-induced apoptosis in human alveolar epithelial cells [45]. Furthermore, Nrf2 activation has been reported to induce caspase-independent apoptosis and inhibit the growth of respiratory infections [153]. Sulforaphane is a naturally occurring isothiocyanate found in broccoli that has been shown to induce apoptosis via activating Nrf2 [154]. Isoliquiritigenin (ISL), a chalcone isolated from licorice (Glycyrrhizae Radix) root, is reported to be a natural Nrf2 agonist inhibiting H1N1, HSV-1, and EMCV replication in vitro [155]. Nrf2 activators have been reported to reduce SARS-COV2 viral pathogenesis by inducing the protease SPLI gene, anti-viral mediators such as RIG-1 and INFs, and by inhibiting anti-apoptotic proteins, TRMPSS2, and the NF-κB pathway [156]. Overall, this evidence highlights the role of the Nrf2 pathway in apoptosis during viral respiratory infections.

## 6. The Role of the Nrf2 Pathway in Respiratory Virus-Induced Fibrosis

Fibrosis refers to the development and excessive accumulation of fibrous tissues formed by extracellular matrix (ECM) component-producing myofibroblasts around inflamed or damaged tissue(s), leading to organ malfunctions and host death [157]. Although fibrosis may affect almost all tissues in the body [157], infections by RVs are most often associated with the development of only pulmonary fibrosis or idiopathic pulmonary fibrosis (IPF) [158,159,160,161,162]. Respiratory viral infections, such as influenza, coronavirus, and RSV have been associated with IPF and other severe complications (Table 4) [73,74,78,90,91,92,163,164,165,166,167,168,169].

It has been reported that the deletion of Nrf2 in mice leads to decreased expression levels of antioxidant enzymes and phase II detoxifying enzymes, resulting in more severe bleomycin-induced inflammation and pulmonary fibrosis [170]. Overall, the activation of Nrf2 constitutes a protective mechanism against the promotion and progression of pulmonary fibrosis in respiratory viral infections by addressing multiple pathways involved in fibrosis pathogenesis, including oxidative stress, inflammation, Th1/Th2 balance, EMD, and EMT control (Figure 1). Given its protective functions, enhancing Nrf2 activity through pharmacological agents or dietary compounds holds a potential promise for preventing or treating pulmonary fibrosis, both generally and in the specific context of respiratory viral infections.

## 7. The Role of the Nrf2 Pathway in Autoimmunity Associated with Viral Respiratory Infections

Autoimmunity, also known as autoimmune disease (AID), occurs after a body’s immune response loses self-antigen tolerance, causing the production of autoantibodies that attack the body’s cells. Viral infections have been considered the major inducing factor, with respiratory viral infections contributing the most in triggering and exacerbating AID in genetically susceptible individuals (Table 5) [73,74,78,91,92,171,172,173,174,175,176,177,178,179,180,181,182,183,184].

Notably, an AID induced by RVs and occurring in the airway system is known as an autoimmune respiratory disease (AIRD). Even though the mechanisms underlying the involvement of RVs in autoimmunity development are still elusive, at least four (4) pathogenic mechanisms, including molecular mimicry [171,172], bystander activation [172,173], dysregulated immune response [174,175], and epitope spreading, [176,177] have been unanimously proposed to explain respiratory virus-induced autoimmunity. The Nrf2 pathway has an established role in the pathogenesis of autoimmunity (Figure 1) [189,190]. The antioxidant and detoxification effects elicited by activating the Nrf2 signaling pathway may serve as a potential defensive mechanism against autoimmunity triggered by environmental pathogens, including RVs [191]. However, this area necessitates further research.

## 8. The Role of the Nrf2 Pathway in Vascular Injury during Viral Respiratory Infections

Respiratory viral infections have been listed among the highly morbid etiological risk factors associated with vascular disease. CoVs (SARS-CoV and SARS-CoV-2) and IVs are the main RVs related explicitly to vascular disease occurrence. It has been reported that COVID-19 patients of all ages are susceptible to undergoing virus-caused endothelial injury and dysfunction [192,193]. SARS-CoV-2 infection both exacerbates a preexisting vascular disease, such as coronary artery disease, and provokes the development of new pathological conditions in immunocompromised patients, such as venous thromboembolism. Similar to human studies, studies on infected animal models reported endothelial damage and vascular thrombosis in the lungs of rhesus macaques infected by SARS-CoV-2 [194]. It has been hypothesized that SARS-CoV-2 triggers the immune response and induces inflammation with the production of ROS, which can cause endothelial damage and dysfunction, impaired vasoregulation, increased permeability-associated vascular leakage, and coagulopathy, which is known to contribute to the development of vascular disease, such as vasculitis, thrombosis, and cardiovascular complications [192,195]. The dysregulation of coagulation pathways and endothelial activation play crucial roles in SARS-CoV-2-induced thrombosis [196].

Armstrong et al. [197] reviewed the role of influenza in the exacerbation of vascular disease similar to SARS-CoV-2. Specifically, infection with IVs induces a pathogenic cytokine storm characterized by elevated cytokines and chemokines (TNF, IL-6, and IL-1β), which upregulate trypsin and result in the loss of the endothelial tight junction protein, zonula occludens-1 (ZO-1), and subsequent vascular hyperpermeability [197,198]. Moreover, a study on the H5N1 influenza strain reported that high and severe H1N1 infection induced elevated chemokine expression, causing endothelial barrier dysfunction and reversely endothelial activation, the loss of barrier function, and microvascular leakage, promoting the severity of the influenza infection. Additionally, several pieces of evidence demonstrate that IV-associated endothelial dysfunction is a risk factor for the occurrence of thrombotic events (reviewed in [197]).

Overall, RVs can induce vascular disease via mechanisms including the direct viral invasion of endothelial cells, the dysregulation of the immune response, and the activation of coagulation pathways.

The activation of the Nrf2 pathway also has anti-inflammatory effects on vascular tissues. Notably, Nrf2 activation suppresses the expression of pro-inflammatory cytokines and adhesion molecules, thereby attenuating endothelial dysfunction and vascular inflammation [199]. Additionally, Nrf2 activation can inhibit the nuclear factor-kappa B (NF-κB) signaling pathway, a central mediator of inflammation in vascular diseases [200,201]. The pharmacological activation of Nrf2 has been shown to attenuate oxidative damage, inhibit inflammation, improve endothelial function, and reduce atherosclerotic lesion formation in preclinical studies [200,202,203,204]. The Nrf2 pathway plays a significant role in protecting against vascular diseases induced by RVs by upregulating antioxidant and anti-inflammatory responses and promoting the transcription of cytoprotective genes, which restore vascular tissue homeostasis and protect against subsequent vascular damage (Figure 1).

## 9. Therapeutic Potential of Nrf2 Activation

As aforementioned and previously discussed [205], Nrf2 plays a crucial role in protecting against respiratory virus-induced injuries through its activation, as it upregulates antioxidant and anti-inflammatory genes, attenuates the replication of RVs (in most respiratory viral infections), and promotes airway tissue and organ repair (Figure 1). The inactivation or dysregulation of the Nrf2 pathway by viruses is associated with viral escape, the enhancement of oxidative stress and lipid peroxidation, and increased susceptibility to respiratory virus-induced injuries, which indicates that induced Nrf2 activation could be a potential therapeutic strategy in respiratory virus-induced injuries. Thus, to obtain the full therapeutic benefits of Nrf2 during respiratory viral infections and to prevent the virus-induced downregulation of Nrf2, researchers have focused on developing Nrf2 activators (known as Nrf2 agonists) and studying their efficacy (and safety) in preclinical and clinical studies (Table 6) [35,37,39,44,45,51,139,155,206,207,208,209,210,211,212,213,214,215,216,217,218,219,220,221,222,223,224,225,226,227,228,229,230,231,232,233,234,235,236,237,238,239].

Multiple therapeutic approaches targeting Nrf2 activation have been investigated in vitro and in vivo. Among the promising molecules for targeting the Nrf2 pathway, natural compounds are widely investigated because they combine the potential to modulate multiple targets simultaneously and due to their safety, availability, and affordability [240,241,242,243,244,245].

Most discovered and investigated activators have shown promising results in preclinical studies and hold great potential for treating RV infections and respiratory virus-induced injuries and complications (Table 6).

Sulforaphane has proven efficacy in reducing symptom severity associated with respiratory virus infections by activating the Nrf2 pathway, and it has also demonstrated positive outcomes in clinical trials. An advanced phase 4 clinical trial is ongoing to confirm the capability of sulforaphane as an Nrf2 activator in reducing respiratory virus replication and the associated complications in the general population. Similarly, curcumin and resveratrol have shown strong Nrf2 activating properties and have demonstrated potential in epigallocatechin gallate. Specifically, curcumin has been demonstrated to inhibit the replication of RSV, influenza, and parainfluenza, thereby reducing the oxidative stress induced by viral infection, possibly via HO-1 activation. Resveratrol, on the other hand, has been found to inhibit the replication of RSV and decrease inflammation in the lungs through Nrf2 activation [205,246,247,248,249,250,251].

Dimethyl fumarate (DMF), an FDA-approved treatment for multiple sclerosis (MS) and psoriasis and a well-known Nrf2 activator, has also shown significant therapeutic potential as it displays an antiviral effect against SARS-CoV-2. Furthermore, DMF has been found to reduce inflammation, restore cell redox homeostasis, and improve lung function in respiratory SARS-CoV-2- and IAV-induced injuries, making it a promising candidate for future therapeutic interventions [25,35,206,252,253]. Bitopertin, a novel 7,7-dioxo-2-thioxo-3-bis(3-quinolinyl) isochromanyl sulfonamide medication used to promote the treatment of hypertension, counts among the promising Nrf2 activators. Current research indicates that bitopertin could reduce inflammation and enhance the body’s defense against oxidative stress in the lungs of animals treated with bitopertin. As a result, these findings suggest that bitopertin may hold promise as a treatment for diseases primarily associated with a decrease in Nrf2, such as lung injuries caused by viruses [228,229,230]. Tempol, a well-known antioxidant agent that activates Nrf2, has been studied for its anti-inflammatory and antifibrotic effects using a mouse model that simulates stimulus-induced conditions. It has been discovered that intervention with Tempol can suppress the production of reactive oxygen species (ROS), inflammatory responses, and cell death and improve organ function [231,232,233,238].

Moreover, besides the cytoprotective effects of Nrf2 pathway activation, Nrf2 agonists may inhibit viral replication. Recently, Waqas et al. showed that Nrf2 agonists such as 4-octyl itaconate (4OI), bardoxolone methyl (BARD), sulforaphane (SFN), and the inhibitor of exportin-1 (XPO1)-mediated nuclear export selinexor (SEL) block IAV replication [254]. Similarly, as shown in Table 6, many other Nrf2 activators may interact with and prevent RV entry. The mechanism by which these agonists would block viral entry and replication remains unclear.

These findings suggest that these drugs could be a promising therapeutic option for treating respiratory virus-induced injuries and preventing associated complications as they offer twofold benefits. However, further research is needed to determine the optimal dosage, administration route, and long-term safety of each of these Nrf2 activators in the context of respiratory viral infections in humans. Additionally, studies investigating the potential synergistic effects of combining different Nrf2 activators for enhanced therapeutic outcomes are warranted. For example, combining sulforaphane, curcumin, and resveratrol may have additive or synergistic effects in activating the Nrf2 pathway and reducing respiratory virus-induced injuries. Further research is thus needed to explore the potential benefits and safety of combining these Nrf2 activators and understand the mechanisms underlying their synergistic effects. Note, however, that most evidence supporting Nrf2 agonists for viral infection treatment comes from in vitro studies and little evidence comes from in vivo validation. These studies often overlook Nrf2 pathway regulation balance; the dysregulation of this pathway can potentially lead to cancer cell proliferation alongside overactivation. Even though in vivo experiments in mice and human trials with sulforaphane show promising outcomes, caution is needed due to potentially harmful effects from NRF2 overactivation. Thus, a further study is essential before therapeutically utilizing Nrf2 agonists alone or in combination.

## 10. Concluding Remarks

Respiratory virus infections are one of the main leading causes of airway damage. Respiratory virus-associated injuries include oxidative stress, ferroptosis, inflammation, pyroptosis, apoptosis, fibrosis, autoimmunity, and vascular injury. The inhibition or deregulation of the Nrf2 pathway activation induces airway tissue damage in viral respiratory infections. In contrast, Nrf2 pathway activation demonstrates protection against tissue and organ injuries. Clinical trials of Nrf2 agonists are needed to define the effect of Nrf2 therapeutics on airway tissues and organs damaged by viral respiratory infections.

## Figures and Tables

**Table 1:** Impact of respiratory viruses on redox and ferroptosis pathways.

Viruses	Impact on Ferroptosis	Reference
Influenza	▪ ↓ in cellular concentration of GSH and/or ↓ in GPX4 activity and ↓ in redox state and normal antioxidant response have been reported in infections with IVs (IAV, swine influenza virus)	[73,74,75,76,77]
	▪ Typical changes in iron metabolism, lipid peroxidation, selenoprotein and GSH levels, and mitochondrial and lysosomal activity have been associated with severity of influenza infections	[75,76,77,78]
SARS-CoV-2	▪ ↑ in oxidative stress, which plays a major role in SARS-CoV-2 infection-induced multiple organ failure	[78]
	▪ ↑ in iron metabolism dysfunction	[79,80]
	▪ ↑ in production of lipid peroxidation markers, such as oxidized phospholipids and 4-hydroxynonenal (HNE)	[81,82]
	▪ ↑ in acyl-CoA synthetase long-chain family member 4 (ACSL4)	[83]
	▪ ↓ in levels of L-cysteine (a rate-limiting precursor of GSH)	[84,85,86,87,88,89]
	▪ ↓ in GSH correlating with ↓ in vitamin D binding protein (VDBP) and VD levels; ↑ in ROS and oxidative stress levels	[78]
	▪ COVID-19 patients show imbalanced iron metabolism causing increased ferritin concentration in blood, which is transferred into cells by TfR1 (transferrin receptor 1), activating Fenton reaction	[78]
RSV	▪ Study on RSV-infected mice described ↑ secretion of pro-inflammatory chemokines CCL5 and CCL3 and ↑ expression of mitochondrial iron content and 12/15-lipoxygenase (12/15-LOX, ↑ deoxygenation of poly unsaturated fatty acids), which correlated with ↑ in 12/15-LOX signaling pathway	[90]
Enterovirus	▪ During enterovirus infections, Coxsackie virus causes ↑ in serum iron intake from gastrointestinal track, which results in ↑ in typical cellular oxidative stress that damages myocardium of mouse models	[78,91,92]

Abbreviations: IAV: influenza A virus, ROS: reactive oxygen species, RSV: respiratory syncytial virus.

**Table 2:** Impact of respiratory viruses on inflammation and pyroptosis.

Viruses	Mechanism of Inflammation	Reference
Common respiratory viruses (RSV, HRV, CoVs, IVs, Other viruses)	▪ ↑ pro-inflammatory cytokines, and chemokines induced by myeloid cells that alter local airway niche and activate immune and non-immune cell inflammation. ▪ ↑ Type I (IFNα/β) and type III (IFNλ) interferons, interleukins (IL)-6, IL-8, IL-12, RANTES, macrophages-associated inflammatory protein 1α and monocytes-associated chemotactic protein 1, in host epithelial cells.	[105,106,107,108,109,110,111,112,113,114,115,116,117,118,119,120,121]
	▪ ↑ innate immune cell infiltration, responsible for production of type II interferon (IFNγ), IL-2, IL-4, IL-5, IL-9, and IL-12.	[105]
	▪ ↑ redox-mediated inflammasome activation → ↑ caspase-1, → ↑ cleavage of gasdermin D (GSDMD) → ↑ regulated form of cell death called pyroptosis → DNA fragmentation and rapid plasma membrane permeability. ▪ ↑ IL-1b, IL-18 → ↑ leukocyte innate immune cell infiltration ▪ ↑ inflammasome activation, including NLRP3 (CoVs, PIVs, and IVs). ▪ In asthmatic patients infected with HRV and RSV, the activated Th2 immune response is biased and ↑ production of IL-4, IL-5, IL-13, RANTES and eotaxin and ↑ in eosinophilic infiltration.	[106,109,110,111,112,113,114,115,116,117,118,119,120,121,122,123]
	▪ ↑ phosphorylation of the redox-sensitive PKC ↑ NRF2 dissociation from KEAP1.	[21,36,43]
Influenza	▪ ↑ pyroptosis-related respiratory epithelium damage	[106,108,109,124,125]
	▪ ↑ proinflammatory responses in endothelial cells and damage in epithelial-endothelial tight junctions	[108,126]
SARS-CoV-2	▪ ↑ epithelial cell inflammation, which is a central cause of lung tissue damage and COVID-19 severity	[22,23,25,27,62,101,120,121,127,128]
	▪ ↑ caspase-1, → ↑ cleavage of gasdermin D (GSDMD) → regulated form of cell death called pyroptosis → DNA fragmentation and rapid plasma membrane permeability.	[120,121,123,129,130]
RSV	▪ ↑ viral bronchiolitis and pneumonia in infants and children	[30,34,35]

Abbreviations: RSV: respiratory syncytial virus, HRV: human rhinovirus, CoVs: coronaviruses, IVs: Influenza viruses.

**Table 3:** The impact of respiratory viruses on the apoptosis pathway.

Viruses	Impact on Apoptosis	Reference
Influenza	▪ Inflammatory response induced by IAV infection causes respiratory epithelial cell death (apoptosis) ▪ In the initial infection phase, IAV ↑ viral products (genes and proteins) that ↓ the pro-apoptotic p53 pathway and ↑ the anti-apoptotic phosphoinositide-3-kinase-protein kinase B (PI3 K-AKT) pathway to ↓ apoptosis-based viral clearance ▪ In the later phase of the infection IAV products ↓ the PI3 K-AKT pathway and ↑ the p53 pathway in order to abruptly lyse cells, spread the infection to neighbor cells and ↑ airway tissue damages ▪ ↑ Fas expression ▪ ↓ PKR and apoptosis ▪ Apoptosis plays a role in viral release	[73,74,106,124,125,141,142]
SARS-CoV-2	▪ SARS-CoV-2 ↑ both intrinsic and extrinsic apoptosis pathway activation to escape antiviral immune response and promote its spread and survival.	[143,144]
	▪ Viral products involved in regulation of SARS-CoV-2 replication and apoptosis dysregulation include but not limited to ORF3α, ORF8, z-VAD-fmk, and CD95/Fas/APO-1.	[145]
	▪ SARS-CoV-2 ORF3α ↑ cleavage-based ↑ of caspase-8, known as a hallmark of the extrinsic apoptotic pathway and also in enhancement of cell death and tissue damage.	[146,147]
	▪ SARS-CoV-2 ORF3α knockdown fails to activate apoptosis and inhibit SARS-CoV-2-associated tissue injuries	[147]
RSV	▪ ↑ interferons and caspase 1	[73]
	▪ Experimental studies have shown that autophagy plays a very crucial role in RSV replication. ▪ ↑ autophagy by ↑ the ROS-AMP-activated protein kinase/mammalian target of rapamycin (AMPK-MTOR) signaling pathway, which in turn ↑ cell apoptosis, responsible for immune cell infiltration, alveolar thickening, and hemorrhage in the lungs.	[148]
Adenoviruses	▪ ↑ apoptosis: ↑ sensitivity to TNFa that induces apoptosis, ↑ PP2A, ↑ p53 ▪ ↓ apoptosis through several mechanisms: interacts with FADD ↓ CD95-mediated apoptosis, ↓ phospholipase A2, ↓ Fas, ↓ p53, ↓ pro-apoptotic proteins of the Bcl-2 family, such as Bax, Bak, BNIP3 and Bnip3L ▪ ↓ apoptosis of the host cell in order to ↑ efficiently and the capacity of the virus to ‘hijack’ host cell apoptotic machinery	[73,74,149,150]
Rhinovirus, enteroviruses	▪ ↑ apoptosis through unknown mechanism	[73]
Coronaviruses	▪ ↑ apoptosis through ORF proteins and unknown mechanisms	[73]

Abbreviations: IAV: Influenza A virus, HO-1: ORF: Open Reading Frame, PKR: protein kinase R, p53: tumor protein 53, PP2A: Protein Phosphatase 2A, ROS: Reactive oxygen species, RSV: Respiratory syncytial virus, TNF-a: Tumor necrosis factor-alpha.

**Table 4:** Impact of respiratory viruses on fibrosis pathways.

Viruses	Impact on Fibrosis	Reference
Respiratory viruses	▪ Severe chronic (but not acute) respiratory viral infections have been associated with IPF. ▪ Acute respiratory viral infections may exacerbate/facilitate, or be exacerbated by pre-existing IPF condition, although the potential role of respiratory viral infection and the pathogenesis mechanism that leads to IPF remain elusive ▪ respiratory viral infections ↑ a pathogenic chronic hyper-inflammatory response by ↑ ROS production and disrupting cell redox homeostasis ▪ ↑ respiratory viral infections ↑ mitochondrial and endoplasmic reticulum stress are involved in the pathogenesis of IPF	[163,164,165]
Influenza	▪ 2009 H1N1 infection-induced ARDS, causes severe lung damage through an activated TGF-β/Smad pathway and ↑ endoplasmic reticulum stress, could promote IPF. ▪ Avian influenza viruses (H7N9 and H5N1) are involved in the occurrence of pulmonary fibrosis ▪ H1N1 ↑ TGF-β expression and activation of the Smad system ▪ H5N1 ↑ TNF-α, FGF, and EGF, fibroblast proliferation, and collagen accumulation and ECM deposition	[73,74,166,167,168]
SARS-CoV-2	▪ More than 30% of patients who survived from severe COVID-19 pneumonia developed IPF six months after being discharged from the hospital ▪ SARS-CoV and MERS-CoV outbreaks have been associated with substantial post viral fibrosis and physiological impairment. ▪ CoVs (MERS-CoV and SARS-CoV) are involved in the occurrence of pulmonary fibrosis ▪ CoVs ↑ pro-inflammatory cytokine storm and expression of type I/III collagen	[78,164,168,169]
RSV	▪ RSV infection promotes IPF through the unfolded protein response in a bleomycin-induced pulmonary fibrosis animal model	[90,165]
Enterovirus	▪ During enterovirus infections, Coxsackie virus ↑ serum iron intake from gastrointestinal track, which ↑ a typical cellular oxidative stress that damages the myocardium of mouse models	[78,91,92]

**Table 5:** Impact of respiratory viruses on autoimmunity.

Viruses	Impact on Autoimmunity	Reference
Respiratory viruses	Mechanisms that mediate respiratory viruses-induced autoimmunity ▪ Molecular mimicry ▪ Bystander activation: respiratory viruses can cause significant and extensive tissue damage that leads to self-antigen release, which might activate autoreactive sentinel CD4+/CD8+ T-cells ▪ Dysregulated immune response ▪ Epitope spreading: during respiratory viral infections, immune response may expand to other specific antigen regions (that were not initially recognized), and lead to autoreactive T-cells and autoimmune response activation	[171,172,173,174,175,176,177]
Influenza	▪ IVs have been involved in the development of Guillain-Barré syndrome (GBS) and associated with molecular mimicry and bystander activation mechanism ▪ IVs has been associated with development of type 1 diabetes (T1D) in genetically predisposed people	[73,74,178,179,180,181,182,183,184]
SARS-CoV-2	▪ CoVs including SARS-CoV-2, SARS-CoV, and MERS-CoV have been associated with several autoimmunity process including SLE, rheumatoid arthritis, and autoimmune thyroiditis through molecular mimicry, bystander activation, or dysregulated immune response	[78,185,186,187,188]
RSV	▪ RSV infection has been associated with development of type 1 diabetes (T1D) in genetically predisposed people	[90,179,181,182,183,184]
Enterovirus	▪ During enterovirus infections, Coxsackie virus ↑ serum iron intake from gastrointestinal track, which ↑ a typical cellular oxidative stress that damages the myocardium of mouse models	[78,91,92]

**Table 6:** NRF2 agonists with protective effects in respiratory viruses-induced injuries.

NRF2 Agonists	Activity of Nrf2 Agonists in Respiratory Viruses-Induced Injuries	References
4-OI and derivatives	▪ ↓ replication of SARS-CoV-2 and IAV ▪ ↓ Interferon responses and inflammation in IAV infection	[35,206,207,208]
DMF	▪ ↓ replication of SARS-CoV-2 ▪ ↓ inflammation in SARS-CoV-2 infection	[35,206]
Curcumin	▪ ↓ replication of IVs, PIVs, and RSV ▪ ↓ oxidative stress through HO-1 activation ▪ ↓ inflammation and lung injury	[51,209,210,211,212]
EGCG	▪ ↓ replication of SARS-CoV-2 and IVs through entry blockage ▪ ↓ damages induced by respiratory viruses	[44,213]
Carbocistein	▪ ↓ TNF-α-induced airway inflammation through suppressing NF-κB and ERK1/2 MAPK pathways	[214,215]
BHBA	▪ ↓ RSV-induced oxidative stress ▪ ↓ sodium arsenite (As(III))-induced cytoxicity in lung epithelial cells and prevent lung cancer	[139,216]
Sulforaphane	▪ ↓ hyperoxia-induced lung inflammation in neonatal mice ▪ ↓ RSV, IAV, and SARS-CoV-2 replication and associated inflammatory cytokines ▪ ↓ oxidative stress and respiratory viral replication ▪ ↑ antifibrosis effects in IPF fibroblasts even under TGF-β stimulation ▪ ↓ DEPs-stimulated inflammation in airway epithelial cells	[37,39,217,218,219,220,221,222,223]
tBHQ	▪ ↑ Nrf2 activation hampered by RSV ▪ ↓ lung injury via regulating macrophage polarization and ARDS ▪ ↑ pro- and anti-inflammatory balance	[31,218,224]
Resveratrol and Isoform γ-tocotrienol	▪ ↓ oxidative stress in the airway epithelium ▪ ↓ LPS-induced ARDS ▪ ↓ lung injury	[45,218,225,226,227]
CDDO-Im and its analogue	▪ ↓ lung injury in hyperoxia and aspiration-induced ARDS	[217,218]
Emodin, Quercetin, and Bitopertin	▪ ↑ antioxidant defense ▪ ↑ inflammation and fibrotic lung injuries	[217,228,229,230]
Tempol and oligonol	▪ ↑ Nrf2-induce antioxidant defense and ▪ ↓ viral replication and inflammation ▪ ↓ ROS-associated injuries	[231,232,233,234,235,236,237,238]
Macrolides (Rapamycin, Metformin)	▪ ↑ expression of antioxidant proteins → ↓ ROS ▪ ↓ resistance of lung adenocarcinoma	[239]
ISL	▪ ↓ H1N1, HSV-1, and EMCV replication and associated complication.	[155]

Abbreviations: ARDS: acute respiratory distress syndrome, IAV: Influenza A virus, IVs: Influenza viruses, PIVs: Para-Influenza viruses, BHA: Butylated hydroxyanisole, DMF: Dimethyl fumarate, EGCG: Epigallocatechin gallate, HO-1: Heme Oxygenase 1, IL-6: Interleukin 6, NRF2: Nuclear factor erythroid 2-related factor, 4-OI: 4-octyl itaconate, RSV: Respiratory Syncytial Virus, SARS-CoV-2: severe acute respiratory syndrome-coronavirus 2, tBHQ: tert-butylhydroquinone, ISL: Isoliquiritigenin.
